# The efficacy of minimally invasive coronary artery bypass grafting (mics cabg) for patients with coronary artery diseases and diabetes: a single center retrospective study

**DOI:** 10.1186/s13019-024-02717-8

**Published:** 2024-04-18

**Authors:** Zhao Guangxin, Chi Liqun, Liang Lin, Liu Jiaji, Ma Xiaolong, Zhang Yuxiao, Huang Qiuyue, Kong Qingyu

**Affiliations:** Department of Cardiac Surgery, Beijing Anzhen Hospital, Capital Medical University, 100029 Beijing, P.R. China

**Keywords:** Cardiothoracic surgery, Minimally invasive cardiac surgery coronary artery bypass grafting (MICS CABG), Diabetes, Coronary atherosclerotic heart disease

## Abstract

**Background:**

conventional coronary artery bypass grafting (CCABG) tends to cause severe complications in patients with comorbid Coronary Artery Diseases (CAD) and diabetes. On the other hand, the Minimally Invasive Cardiac Surgery Coronary Artery Bypass Grafting (MICS CABG) via transthoracic incision is associated with rapid recovery and reduced complications. Adding to the limited literature, this study compares CCABG and MICS CABG in terms of efficacy and safety.

**Methods:**

Herein, 104 CCABG and MICS CABG cases (52 cases each) were included. The patients were recruited from the Minimally Invasive Cardiac Surgery Center, Anzhen Hospital, between January 2017 and December 2021 and were selected based on the Propensity Score Matching (PSM) model. The key outcomes included All-cause Death, Myocardial Infarction (MI), Cerebrovascular Events, revascularization, Adverse Wound Healing Events and one-year patency of the graft by coronary CTA.

**Results:**

Compared to CCABG, MICS CABG had longer surgical durations [4.25 (1.50) h vs.4.00 (1.13) h, *P* = 0.028], but showed a reduced intraoperative blood loss [600.00 (400.00) mL vs.700.00 (300.00) mL, *P * = 0.032] and a lower secondary incision debridement and suturing rate (5.8% vs.19.2%, *P* = 0.038). In follow up, no statistically significant differences were found between the two groups in the cumulative Major Adverse Cardiovascular and Cerebrovascular Events (MACCEs) incidence (7.7% vs. 5.9%), all-cause mortality (0 vs. 0), MI incidence (1.9% vs. 2.0%), cerebral apoplexy incidence (5.8% vs. 3.9%), and repeated revascularization incidence (0 vs. 0) (*P* > 0.05). Additionally, coronary CTA results revealed that the two groups’ one-year graft patency (94.2% vs. 90.2%, *P* = 0.761) showed no statistically significant difference.

**Conclusion:**

In patients with comorbid CAD and diabetes, MICS CABG and CCABG had comparable revascularization performances. Moreover, MICS CABG can effectively reduce, if not prevent, poor clinical outcomes/complications, including incision healing, sternal infection and prolonged length of stay in diabetes patients.

## Introduction

Coronary Artery Bypass Grafting (CABG) is better than Percutaneous Coronary Intervention (PCI) in treating patients with comorbid Coronary Artery Diseases (CAD) and diabetes [1, 2]. According to research, CAD are often complex when accompanied by Type II diabetes, with patients’ coronary angiographic characteristics generally manifesting as small, diffuse, calcified, and multi-vessel lesions [3, 4]. But CABG clinical outcomes are often worse in diabetes patients than in non-diabetes patients. There is a greater incidence of incision infection, ischemic cardiovascular events, and neurological and renal complications in diabetes patients, which might explain the high mortality rates [5, 6]. Furthermore, the degree of myocardial ischemia in diabetes patients and the body’s sensitivity to systemic blood circulation changes are usually more significant than those in non-diabetes patients due to coronary angiographic characteristics in patients with comorbid CAD and diabetes.

Minimally Invasive Cardiac Surgery Coronary Artery Bypass Grafting (MICS CABG) has several advantages over the Conventional coronary artery bypass grafting (CCABG) via transthoracic incision, including quick recovery, minimal bleeding, and zero risk of sternal infection [7]. In 1994, Benetti from Buenos Aires was the first to perform a Left Internal Mammary Artery (LIMA) bypass graft to the left anterior descending (LAD) artery on the beating heart through a left sided mini-thoracotomy and with the use of thoracoscopy [8]. After that, various minimally invasive and robotic coronary artery bypass grafting operations have been carried out since 1996; Until 2019, six major surgical approaches are adopted and recognized by the world, and MICS CABG is one of them [9]. Several medical centers have recently reported the efficacy of MICS CABG, with promising clinical results, and its graft patency is comparable to CCABG [10, 11]. Additionally, MICS CABG with the bilateral Internal Mammary Artery (BIMA) showed favourable early outcomes and early graft patency [12]. Herein, we retrospectively investigated patients with comorbid CAD and diabetes who underwent either MICS CABG or CCABG based on the Propensity Score Matching (PSM) model, focusing on one-year graft patency, all-cause mortalities, and incidences of Major Adverse Cardiovascular and Cerebrovascular Events (MACCEs) as the key outcomes. In this regard, the efficacy and safety of MICS CABG in treating patients with comorbid CAD and diabetes were evaluated.

## Experimental

### Clinical method

This study was ethically approved by the Ethics Committee of Beijing Anzhen Hospital, Capital Medical University, China.

A total of 639 bypass operations were conducted in the Minimally Invasive Cardiac Surgery Center, Beijing Anzhen Hospital, China, between January 2017 and December 2021.

Our study included some of these bypass operations, and the inclusion criteria were as follows: (1) Patients with a history of Type II diabetes lasting one year or more; (2) Patients diagnosed with single, multiple, or left main CAD through coronary angiography; (3) Patients with complete clinical data. The exclusion criteria were as follows: (1) Patients with valve diseases, ventricular aneurysms, or other diseases requiring simultaneous cardiac surgery; (2) Patients suffering from severe lung disease who require simultaneous lung resection surgery; (3) Patients with carotid stenosis requiring simultaneous carotid artery stent surgery and emergency operation [13, 14]. After selection, 173 cases were enrolled (65 MICS CABG and 108 CCABG cases, respectively). The cases were matched based on gender, age, Body Mass Index (BMI, kg/m^2^), body surface area (m^2^), history of hypertension, smoking history, drinking history, history of Atrial Fibrillation (AF), history of cerebral infarction, history of cardiac surgery, heart function grading (NYHA grading), creatinine clearance rate (mL/min), brain natriuretic peptide (BNP, ng/L), Creatine Kinase Isoenzyme (CK-MB, ng/mL), Cardiac Troponin (TnI, µg/L), arterial partial pressure of oxygen (PO_2_) before operation, and preoperative Left Ventricular Ejection Fraction (LVEF, %). Finally, 52 MICS CABG cases and 52 CCABG cases were successfully matched. Table 1 summarizes the preoperative characteristics of the enrolled patients.

### Surgery and perioperative period treatment methods

Patients in the CCABG group were placed in a supine position. Subsequently, under general anaesthesia, the sternum was split in the middle through sternotomy incision, gradually spreading the sternum and releasing the Left Internal Mammary Artery (LIMA). The pericardium was not opened at this point. Simultaneously, the Saphenous Vein Graft (SVG) was prepared for backup. The pericardium was opened and suspended after completing the transplantation of blood vessels. The target vessel was fully exposed using a cardiac stabilizer. End-to-side anastomosis was performed on LIMA to the anterior descending artery, while SVG sequential anastomosis was performed on other target vessels. During proximal anastomosis, aortic sidewall forceps were employed on the ascending aorta with no or mild calcification. The ascending aorta with severe calcification was punched with an aortic connector. A flow meter was used to detect graft flow after each target vessel’s revascularization.

Patients in the MICS CABG group were also placed in a supine position and their left shoulder-back side was raised by 30°. Subsequently, general anesthesia was administered, and a dual lumen tracheal intubation was performed to assist the patients with breathing. Following that, the pleura was accessed through the 4th or 5th intercostal space on the left side after making an incision on the anterolateral side of the left chest wall. An IMA retractor (THORATRAK MICS Retractor System) was used to retract the left rib, and the patients were then ventilated with one lung to obtain free access to the LIMA while preparing SVG for backup. Following that, the pericardium was opened and suspended after opening the intercostal space on both sides using a hinged retractor (ValveGate). A cardiac surface fixator (TS2000 Medtronic Inc) was applied to complete anastomosis from the LIMA to the anterior descending artery. The anastomosis site of the proximal ascending aorta was exposed. Proximal venous anastomosis was performed using a specially designed ascending aortic wall forceps. Sequential SVG anastomosis was performed using an apex fixator (29,800 Medtronic Inc) and a cardiac surface fixator. The flow meter was used to detect graft flow after revascularization.

Aspirin and clopidogrel were discontinued before the operation as part of the preoperative anti-platelet regimen. Patients who received PCI and were diagnosed with coronary stent stenosis via coronary angiography or left main artery disease before surgery, were treated with a single anti-platelet regimen (100 mg/d aspirin) up until two days before the operation. The patients underwent extubation in the Intensive Care Unit (ICU) after surgery and later received a daily oral anti-platelet regimen of aspirin (100 mg) and clopidogrel (75 mg) after being transferred back to the general ward. The standard anti-platelet regimen for patients after discharge is lifelong aspirin and clopidogrel for at least one year.

According to the previous history of hypoglycemic drugs, the preoperative blood glucose control regimen was to stop the thiazolidinediones and DPP-4 inhibitors, continue to take metformin and alpha-glucosidase inhibitors, and increase the use of SGLT-2 inhibitors hypoglycemic drugs after admission. The target of blood glucose management is 6.0 to 8.0mmol/L for Fasting Blood-Glucose (FBG) before three meals, and strict attention should be paid to avoid hypoglycemia. Patients with persistent FBG ≥ 10.0mmol/L were treated with subcutaneous insulin injection. The patients received a daily oral hypoglycemic drug regimen after being transferred back to the general ward, and Regular Insulin (RI) was applied for at least 3 days to control blood glucose fluctuations. On the 4th day after surgery, it was decided whether to stop RI according to the FBG and blood glucose fluctuations.

Antibiotics were used on the day of surgery, and the conventional antibiotics were second- or third-generation cephalosporin antibiotics.

### Variation definitions

The diagnosis of CAD was confirmed by coronary angiography. Diabetes was defined as typical diabetic symptoms (including polydipsia, polyuria, polyphagia, and unexplained weight loss) accompanied by Random Blood Glucose (RBG) ≥ 11.1mmol/L, FBG ≥ 7.0mmol/L, Oral Glucose Tolerance Test (OGTT) 2 h blood glucose ≥ 11.1mmol/L, or glycosylated hemoglobin (HbA1c) ≥ 6.5%. All-cause mortality was defined as death caused by various reasons, including cardiogenic death in patients who were in stable conditions in the past. Myocardial Infarction (MI) was defined as follows: (1) Myocardial enzymes, such as Cardiac Troponin (TnI) and/or Creatine Kinase Isoenzyme (CK-MB), reaching more than twice the upper limit of the normal reference range; and (2) Significant changes in the ST segment of the electrocardiogram. Revascularization events refer to those performed by PCI or CABG after discharge. New York Heart Association (NYHA) classify the impaired state of heart function into four grades according to the degree of activity that induces symptoms of heart failure. It includes Class I: No limitations. Ordinary physical activity does not cause undue fatigue, dyspnoea or palpitations (asymptomatic LV dysfunction); Class II: Slight limitation of physical activity. Ordinary physical activity results in fatigue, palpitation, dyspnoea or angina pectoris (mild CHF); Class III: Marked limitation of physical activity. Less than ordinary physical activity leads to symptoms (moderate CHF) and Class IV: Unable to carry on any physical activity without discomfort. Symptoms of CHF present at rest (severe CHF). Major Adverse Cardiovascular and Cerebrovascular Events (MACCEs) includes death, nonfatal myocardial infarction, revascularization and cerebral apoplexy. The reference range of the creatinine clearance rate (eGRF) was > 90 mL/L; with 60–90 mL/min, 30–60 mL/min, 15–30 mL/min, and < 15 mL/min indicating minor renal impairment, medium renal impairment, heavy renal impairment, and dialysis, respectively. The reference range of BNP was 0-100 ng/L, with 100–300 ng/L and > 300 ng/L indicating cardiac dysfunction and acute heart failure, respectively. On the other hand, the reference range of PO_2_ was 80–100 mmHg, with 60–80 mmHg and < 60 mmHg indicating mild hypoxemia and Type I respiratory failure, respectively. The three-level FitzGibbon grading standardization (Grade A: Completely unobstructed; Grade B: Stenosis < 50% of the target coronary artery diameter; and Grade O: Graft vessel occlusion) was used to evaluate the transplanted blood vessels, that is, stenosis at the proximal/distal anastomosis or the main stem of the graft.

### Observation index and follow-up

The key outcomes included MACCEs, all-cause death, MI, cerebral apoplexy, revascularization, adverse wound healing events during postoperative follow-up and one-year patency of the graft by coronary CTA. The perioperative complications in the hospital was also recorded.

The contact information of all patients was recorded at discharge, and they were all reminded (together with their families) to undergo coronary artery CTA at our facility or another hospital after one year for graft patency evaluation. we conducted patient follow-up through telephone inquiry, focusing on survival, MACCEs incidence, repeated revascularization incidence and incision healing.

### Statistical analysis

The preoperative confounding factors were filtered based on PSM. Patients were divided into two groups (MICS CABG and CCABG) in a 1:1 ratio based on gender, age, BMI (kg/m^2^), body surface area (m^2^), history of hypertension, smoking history, drinking history, history of AF, history of cerebral infarction, history of cardiac surgery, heart function grading (NYHA grading), creatinine clearance rate (mL/min), BNP (ng/L), CK-MB (ng/mL), TnI (µg/L), PO_2_, and preoperative LVEF (%). The caliper value was set at 0.05 and the Standardized Difference (SD) at < 10% to ensure the variables between the two groups matched appropriately. All statistical analyses were performed using IBM SPSS Statistics® for Windows (v 26.0). Normally distributed measurement data were expressed as mean ± standard deviation, and inter-group comparisons were performed using an independent sample t-test or analysis of variance. Non-normally distributed measurement data were presented as Median (M) and Interquartile Ranges [IQR, upper and lower quartiles (P25, P75)], and the Wilcoxon rank sum test was performed for inter-group comparisons. On the other hand, counting data were expressed as frequency (rate or constituent ratio), and inter-group comparisons were performed using a chi-square test, a Fisher’s exact probability test, or the Wilcoxon rank sum test. Kaplan-Meier survival curves were constructed to evaluate MACCEs incidence, and the log-rank test was employed to compare inter-group data. The impact of confounding variables on MACCEs incidence was minimized using a multivariate Cox proportional hazard regression model. Variables with significant statistical differences (*P* ≤ 0.05) or reported effects on the research results were included in the multivariate Cox proportional hazard regression model for correction. Overall, results with *P* ≤ 0.05 were considered statistically significant.

## Results

### Baseline information

Herein, 173 patients were subjected to the PSM model, after which 52 MICS CABG cases and 52 CCABG cases were successfully matched. The caliper value was set at 0.05, and the matching of the variables between the two groups was balanced. There were no statistically significant differences between the CCABG and MICS CABG groups in baseline characteristics of each item (*P* > 0.05), as shown in Table 1, which summarizes the baseline information of all the 104 cases.

**Table 1:** Baseline information of the two groups [cases (%) / x̄±s ]

Clinical Data		CCABG group (*n* = 52)	MICS CABG group (*n* = 52)	p value
Gender (M)		43 (82.6)	42 (80.8)	0.800
Age (years)		60.83 ± 8.62	61.27 ± 8.94	0.798
BMI (kg/m^2^)		25.91 ± 2.67	25.64 ± 2.95	0.627
Body surface area (m^2^)		1.80 ± 0.17	1.82 ± 0.14	0.586
History of hypertension		35 (67.3)	39 (75.0)	0.387
Smoking history		34 (65.4)	29 (55.8)	0.316
Drinking history		21 (40.4)	18 (34.6)	0.543
History of atrial fibrillation		1 (1.9)	0 (0)	0.315
History of cerebral infarction		18 (34.6)	19 (36.5)	0.838
History of cardiac surgery		0 (0)	0 (0)	-
NYHA grading	Level I	0 (0)	0 (0)	0.873
	Level II	6 (11.5)	5 (9.6)	
	Level III	35 (67.3)	34 (65.4)	
	Level IV	11 (21.2)	13 (25.0)	
Creatinine clearance rate (mL/min)	Normal	33 (63.5)	29 (55.8)	0.712
	Mild	17 (32.3)	21 (40.4)	
	Moderate	2 (3.8)	2 (3.8)	
	Severe	0 (0)	0 (0)	
	Dialysis	0 (0)	0 (0)	
BNP (ng/L)	Normal	44 (84.6)	45 (86.5)	0.523
	Cardiac insufficiency	8 (15.4)	6 (11.5)	
	Acute heart failure	0 (0)	1 (1.9)	
CK-MB (ng/ mL)	Normal	52 (100)	52 (100)	-
	> 2 fold	0 (0)	0 (0)	
TnI (µg/L)	Normal	50 (96.2)	50 (96.2)	1.000
	> 2 fold	2 (3.8)	2 (3.8)	
PO_2_	Normal	42 (80.8)	42 (80.8)	1.000
	Mild hypoxemia	10 (19.2)	10 (19.2)	
	Type I respiratory failure	0 (0)	0 (0)	
LVEF (%)	≥ 50	50 (96.2)	50 (96.2)	1.000
	< 50	2 (3.8)	2 (3.8)	

NYHA, New York Heart Association

### Perioperative outcomes

Compared to the CCABG group, the MICS CABG group’s surgical duration was slightly longer [4.25 (1.50) h vs.4.00 (1.13) h, *P* = 0.028], but with less intraoperative blood loss [600.00 (400.00) mL vs.700.00 (300.00) mL, *P* = 0.032]. Additionally, the length of stay post-surgery was shorter in the MICS CABG group than in the CCABG group[5.00 (1.00) d vs.6.00 (1.00) d, *P* = 0.068], but with no statistically significant difference (Table 2). No statistically significant difference was found between the two groups in intraoperative urine volume, length of ICU stay, postoperative mechanical ventilation, postoperative 24-h bleeding volume, and postoperative blood transfusion treatment (Table 2). Regarding surgery, both groups underwent off-pump coronary artery bypass grafting (OP CABG) and no statistically significant difference was observed in their graft numbers [3.00 (2.00) vs.3.00 (1.00), *P* = 0.734]. Regarding the graft type, the CCABG group had a higher proportion of veins than the MICS CABG group (38.0% vs.2.0%, *P* < 0.001). However, Radial Artery (RA) grafts were used in four patients in the CCABG group, of which one case underwent total arterial vascular transplantation, with a statistically significant difference (*P* < 0.001). No statistically significant difference was found between the two groups in perioperative complications such as mortality, MI, cerebral apoplexy, reoperation, utilization of Intra-Aortic Balloon Pump (IABP), utilization of Extracorporeal Membrane Oxygenation (ECMO) and utilization of Continuous Renal Replacement Therapy (CRRT) (*P* > 0.05, see Table 2). Table 3 summarizes the specific reasons why LIMA was not utilized in patients in the two groups.

**Table 2:** Perioperative data of the two groups [cases (%) / x̄±s ]

Clinical Data		CCABG group (*n* = 52)	MICS CABG group *n* = 52)	p value
Surgical duration (h)		4.00(1.13)	4.25(1.50)	0.028
Intraoperative blood loss (mL)		700.00(300.00)	600.00(400.00)	0.032
Intraoperative urine volume (mL)		1200.00(975.00)	1200.00(1400.00)	0.804
bypass grafts Number		3.00(1.00)	3.00(2.00)	0.734
Postoperative mechanical ventilation (h)		16.00(6.90)	15.50(3.90)	0.873
Length of ICU stay after surgery (h)		18.75(6.90)	19.00(5.00)	0.656
Postoperative 24-h bleeding volume (mL)		300.00(277.50)	290.00(287.50)	0.151
Length of stay after surgery (d)		6.00(1.00)	5.00(1.00)	0.068
Blood transfusion after surgery		2 (3.8)	1 (1.9)	0.558
off-pump coronary artery bypass grafting		52 (100)	52 (100)	-
coronary endarterectomy		0 (0)	3 (5.8)	0.241
Graft type	Veins	20 (38.5)	1 (1.9)	< 0.001
	Left internal mammary + vein	28 (53.8)	51 (98.1)	
	Left internal mammary + radial artery + vein	3 (5.8)	0 (0)	
	Left internal mammary + right internal mammary + radial artery	1 (1.9)	0 (0)	
Perioperative complications	Mortality	1 (1.9)	0 (0)	1
	MI	1 (1.9)	0 (0)	1
	Cerebral apoplexy	1 (1.9)	0 (0)	1
	Reoperation	1 (1.9)	0 (0)	1
	Utilization of IABP	2 (3.8)	0 (0)	0.475
	Utilization of ECMO	0 (0)	0 (0)	-
	Utilization of CRRT	0 (0)	0 (0)	-

**Table 3:** IMA utilization in the two groups

Clinical Data	CCABG group (*n* = 20)	MICS CABG group (*n* = 1)
Fine inner diameter of LIMA (< 0.16 cm)	4	0
Low flow rate of LIMA (< 40 cm/s)	3	0
Insufficient LIMA length after anterior descending stent surgery	3	0
Unsatisfactory material selection of LIMA or dissection	2	1
Subclavian artery stenosis	6	0
Advanced age (> 75 years old)	2	0

LIMA, Left Internal Mammary Artery

### Follow-up

**Fig. 1:**
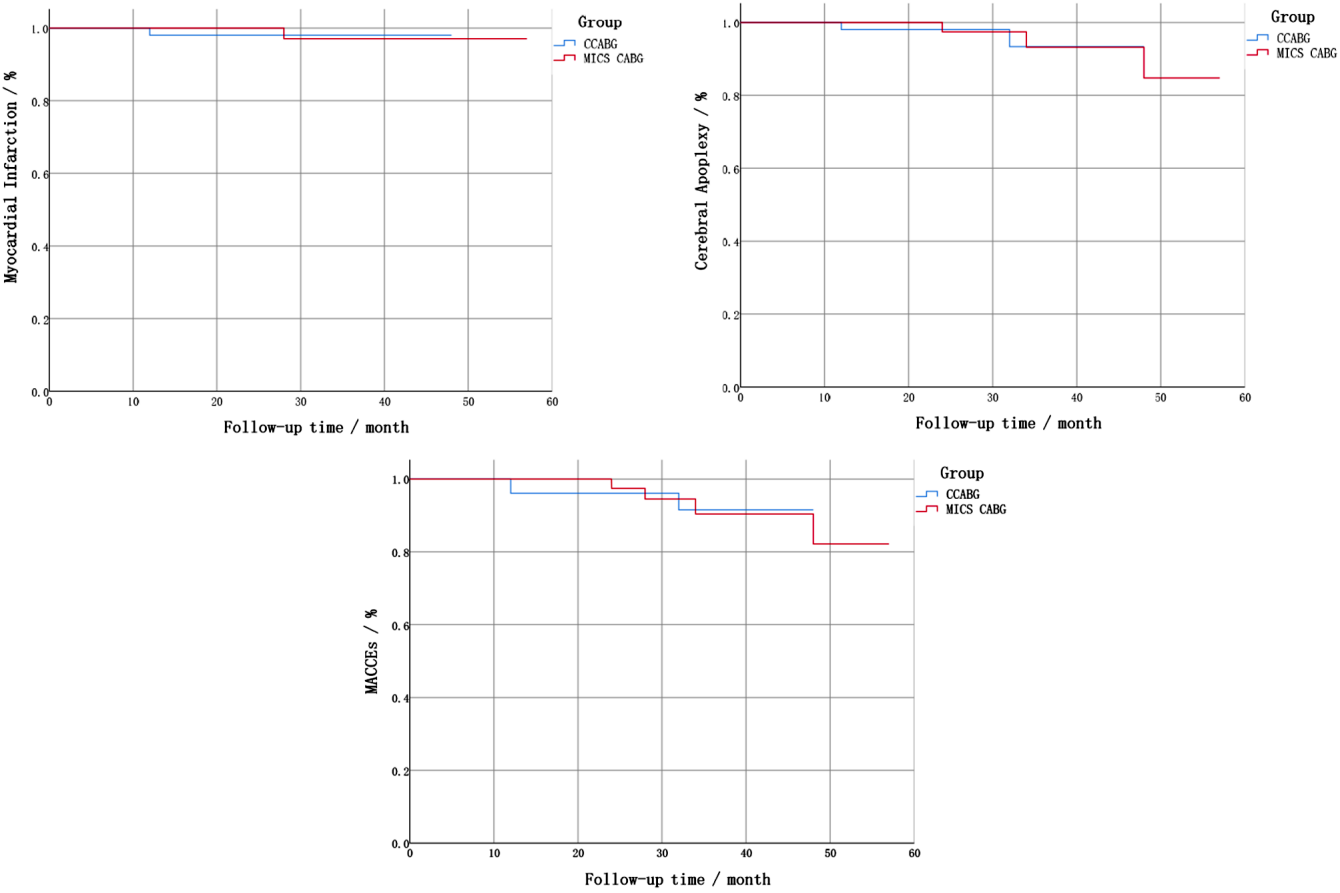
Follow-up results. MACCEs, Major Adverse Cardiovascular and Cerebrovascular Events.

Except for one patient in the CCABG group who died of perioperative MI and cerebral apoplexy, all patients had complete follow-up data. The median follow-up periods of the CCABG and MICS CABG groups were 2.42 and 2.58 years, respectively. Based on Kaplan-Meier curves, there was no statistically significant difference in the cumulative MACCEs incidence, all-cause mortality, MI incidence, cerebral apoplexy incidence and repeated revascularization incidence between the two groups (*P* > 0.05, Table 4; Fig. 1). According to the coronary CTA results, the one-year graft patency of the two groups also showed no statistically significant difference (*P* > 0.05, Table 4). In order to reduce the influence of graft types on the prognosis of two surgical procedures, we selected all 32 patients in CCABG group who used LIMA as the new LIMA CCABG group (L-CCABG). Meanwhile, according to the number of pairs in the Propensity Score Matching model, we correspondingly selected 32 patients in MICS CABG group as the new LIMA MICS CABG group (L-MICS CABG) and conducted subgroup analysis, as shown in Table 5. There was still no statistically significant difference in the cumulative MACCEs incidence, all-cause mortality, MI incidence, cerebral apoplexy incidence, repeated revascularization incidence and one-year graft patency between the two subgroups (*P* > 0.05). To eliminate the impact of suspicious factors on the follow-up results, a multivariate Cox model was constructed to re-correct the basic data of the two groups. According to the results, no statistically significant difference was found in the cumulative MACCEs incidences between the two groups after calibration (*P* > 0.05). However, incision healing was better in the MICS CABG group than in the CCABG group, with the former showing a lower proportion of secondary debridement and suturing caused by incision infection or fat liquefaction (5.8% vs. 19.2%, *P* = 0.038). These differences were statistically significant (Table 4).

**Table 4:** Postoperative coronary CTA and prognosis of the two groups [cases (%)]

Clinical Data		CCABG group (*n* = 51)	MICS CABG group (*n* = 52)	p value
Grading of vein grafts	A	34 (66.7)	38 (73.1)	0.761
	B	12 (23.5)	11 (21.1)	
	O	5 (9.8)	3 (5.8)	
MACCEs		3 (5.9)	4 (7.7)	0.715
All-Cause Mortality		0 (0)	0 (0)	-
Myocardial Infarction		1 (2.0)	1(1.9)	0.989
Cerebral Apoplexy		2 (3.9)	3 (5.8)	0.663
Repeated Revascularization		0 (0)	0 (0)	-
Secondary debridement and suturing		10 (19.2)	3 (5.8)	0.038

MACCEs, Major Adverse Cardiovascular and Cerebrovascular Events

**Table 5:** Postoperative coronary CTA and prognosis of the two LIMA subgroups [cases (%)]

Clinical Data		L-CCABG group(*n* = 32)	L-MICS CABG group (*n* = 32)	p value
Grading of vein grafts	A	23 (71.9)	22 (68.8)	0.580
	B	8 (25.0)	7 (21.8)	
	O	1 (3.1)	3 (9.4)	
MACCEs		1 (3.1)	3 (9.4)	0.302
All-Cause Mortality		0 (0)	0 (0)	-
Myocardial Infarction		0 (0)	1 (3.1)	0.313
Cerebral Apoplexy		1 (3.1)	2 (6.2)	0.554
Repeated Revascularization		0 (0)	0 (0)	-

MACCEs, Major Adverse Cardiovascular and Cerebrovascular Events; L-CCABG, LIMA CCABG group, L-MICS CABG LIMA MICS CABG group

## Discussion

Compared to CCABG, MICS CABG has stricter surgical contraindications, which mainly include severe peripheral artery disease, intolerance to unilateral pulmonary ventilation in Chronic Obstructive Pulmonary Disease (COPD), morbid obesity, thoracocyllosis, and severe calcification of ascending aorta [15]. As a result, we conducted a more detailed preoperative evaluation of the MICS CABG group. Additionally, other factors were used to correct the baseline data of the two groups through PSM. The difficulty of proximal anastomosis at the ascending aorta is generally greater in MICS CABG than in CCABG due to the small incision size and distance from the ascending aorta. Therefore, as recommended in previous research [7, 16], IMA was used in the MICS CABG group to reduce the number of proximal anastomosis, and sequential bridge anastomosis was performed on SVG. Herein, IMA was essential in the preoperative evaluation of MICS CABG. On the other hand, CCABG was selected for patients with poor preoperative IMA evaluation. Thus, the rate of IMA utilization was higher in the MICS CABG group than in the CCABG group in this study.

Incomplete vascularization and graft lesions are common causes of post-CABG recurrence-induced MACCEs. A previous study demonstrated the safety and effectiveness of MICS CABG [14]. Herein, the five-year cumulative MACCEs incidence (all-cause mortality, MI, cerebral apoplexy, and repeated revascularization) was 11.3%. Compared to MICS CABG, revascularization occurrence after CCABG was significantly later, although the interval between the two groups was only 4.4 months and the results were not clinically significant [17]. In our study, no statistically significant differences were also found in all-cause mortalities and MI, cerebral apoplexy, repeated revascularization incidences and one-year coronary graft patency by CTA of the CCABG and MICS CABG groups. It confirms that MICS CABG could achieve a similar degree of revascularization effect as CCABG in diabetes patients.

In a past study, diabetes patients who underwent CABG exhibited a slow recovery of body function [18]. As a result, the physical function of diabetes patients, including effective rehabilitation activities in the early postoperative period and incision healing, merits special attention [19]. At the same time, the post-CABG unfavourable clinical outcomes/complications in diabetes patients, including poor incision healing, sternal infection, cerebral apoplexy, acute renal failure, extended ICU stay, and increased demand for blood transfusion, should be investigated [20]. Furthermore, it is noteworthy that using IMA may increase the incidence of sternal wound complications in diabetes patients, especially those under insulin treatment [21]. Overall, the impact of diabetes on the efficacy of CABG can be deduced as multifaceted.

In contrast to CCABG, MICS CABG does not require sternotomy, which not only reduces postoperative pain but also aids in postoperative expectoration and early rehabilitation training [16, 22]. Herein, compared to patients undergoing CCABG, patients receiving MICS CABG had a less intraoperative blood loss and a shorter length of stay post-surgery. Moreover, the follow-up results revealed that incision healing in diabetes patients was relatively slow in the MICS CABG group. This finding suggests that MICS CABG can effectively reduce, if not prevent, unfavourable clinical outcomes/complications, such as poor incision healing, sternal infection, and increased blood transfusion requirements in diabetes patients.

Due to the small, diffuse, calcified, and multi-vessel lesions usually present in the coronary arteries [3, 4] and the small surgical field in MICS CABG, the overall surgical risks and difficulty in complete revascularization were significantly increased when performing MICS CABG on diabetes patients. However, several methods can still be used to lower surgical risks and ensure patients’ perioperative safety. First, the surgical space could be expanded by extending the incision length and skin incision 2–3 cm outwards. Second, strict quality control should be enforced during the operation, and a vascular flow meter should be used to monitor the flow rate of the transplanted blood vessels. Third, it is necessary to reasonably control the patients’ blood glucose levels after the operation. Low blood glucose levels were found to be correlated with poor cardiovascular prognosis of CAD patients [23, 24]. Following cardiac surgery, appropriate blood glucose level control (7.8–10.0 mmol/L) could help improve clinical outcomes [25, 26]. In a previous study, the fluctuation amplitude of blood glucose levels was significantly correlated with post-CABG adverse events. Strict postoperative control of blood glucose levels (< 7.8 mmol/L) led to significant fluctuations in blood glucose levels [27]. To minimize the incidence of postoperative adverse events, the blood glucose level should be controlled within a relatively appropriate range (7.0–10.0 mmol/L) after MICS CABG and CCABG.

This study has several limitations. First, it is a single-center retrospective study, and the analysis can only be based on existing data and results. Second, there was a certain selection bias when objectively grouping the patients. However, this study selected patients with comorbid CAD and diabetes as objects, and analyzed the safety and efficacy of MICS CABG in them. The results showed that MICS CABG had better efficacy in diabetic patients than expected, as well as better wound healing compared with CCABG. Therefore, a large sample size and more follow-up results are required to verify the medium- and long-term efficacy of MICS CABG in diabetes patients, which we plan to further explore in future studies.

## Conclusions

Herein, all-cause mortalities and MI, cerebral apoplexy, and repeated revascularization incidences of the CCABG and MICS CABG groups exhibited no statistically significant differences. Similarly, no statistically significant difference was found in their one-year graft patency by coronary CTA. These findings collectively suggest that MICS CABG could achieve the same degree of revascularization effect as CCABG. Moreover, despite the increased surgical duration, MICS CABG is characterized by reduced intraoperative blood loss and a low incision secondary debridement and suturing rate. As a result, it can effectively reduce, if not prevent, unfavourable clinical outcomes/complications, including poor incision healing, sternal infection and prolonged length of stay in diabetes patients.

## Data Availability

The datasets used and analyzed during the current study are available from the first author on reasonable request.

## Abbreviations

AF: Atrial Fibrillation
BIMA: Bilateral Internal Mammary Artery
BMI: Body Mass Index
BNP: Brain Natriuretic Peptide
COPD: Chronic Obstructive Pulmonary Disease
CCABG: Conventional Coronary Artery Bypass Grafting
CRRT: Continuous Renal Replacement Therapy
CABG: Coronary Artery Bypass Grafting
CAD: Coronary Artery Diseases
ECMO: Extracorporeal Membrane Oxygenation
FBG: Fasting Blood-Glucose
IABP: Intra-Aortic Balloon Pump
IMA: Internal Mammary Artery
ICU: Intensive Care Unit
IQR: Interquartile Ranges
LIMA: Left Internal Mammary Artery
LVEF: Left Ventricular Ejection Fraction
MACCEs: Major Adverse Cardiovascular and Cerebrovascular Events
MICS CABG: Minimally Invasive Coronary Artery Bypass Grafting
MI: Myocardial Infarction
OGTT: Oral Glucose Tolerance Test
OP CABG: Off-Pump Coronary Artery Bypass Grafting
PO_2_: Partial Pressure of Oxygen
PCI: Percutaneous Coronary Intervention
PSM: Propensity Score Matching
RBG: Random Blood Glucose
RA: Radial Artery
RCA: Right Coronary Artery
RI: Regular Insulin
SVG: Saphenous Vein Graft
